# Benchmarking Brain-Computer Interfaces Outside the Laboratory: The Cybathlon 2016

**DOI:** 10.3389/fnins.2017.00756

**Published:** 2018-01-11

**Authors:** Domen Novak, Roland Sigrist, Nicolas J. Gerig, Dario Wyss, René Bauer, Ulrich Götz, Robert Riener

**Affiliations:** ^1^Sensory-Motor Systems Lab, Department of Health Sciences and Technology, ETH Zurich, Zurich, Switzerland; ^2^Department of Electrical and Computer Engineering, University of Wyoming, Laramie, WY, United States; ^3^Department of Design, Specialization in Game Design, Zurich University of the Arts, Zurich, Switzerland

**Keywords:** benchmark testing, brain-computer interfaces, competition, electroencephalography, machine learning

## Abstract

This paper presents a new approach to benchmarking brain-computer interfaces (BCIs) outside the lab. A computer game was created that mimics a real-world application of assistive BCIs, with the main outcome metric being the time needed to complete the game. This approach was used at the Cybathlon 2016, a competition for people with disabilities who use assistive technology to achieve tasks. The paper summarizes the technical challenges of BCIs, describes the design of the benchmarking game, then describes the rules for acceptable hardware, software and inclusion of human pilots in the BCI competition at the Cybathlon. The 11 participating teams, their approaches, and their results at the Cybathlon are presented. Though the benchmarking procedure has some limitations (for instance, we were unable to identify any factors that clearly contribute to BCI performance), it can be successfully used to analyze BCI performance in realistic, less structured conditions. In the future, the parameters of the benchmarking game could be modified to better mimic different applications (e.g., the need to use some commands more frequently than others). Furthermore, the Cybathlon has the potential to showcase such devices to the general public.

## Introduction

Noninvasive brain-computer interfaces (BCIs), which measure a human's brain activity and use it to control machines, have the potential to improve human-machine interaction in numerous ways. As assistive devices, they can be used by people with disabilities to control wheelchairs (Carlson and Millán, 2013), orthoses (Ortner et al., 2011; Do et al., 2013), neuroprostheses (Rohm et al., 2013) and robots (Leeb et al., 2015) as well as to write messages (Sellers et al., 2014). They can also be used by unimpaired people to play computer games (Coyle et al., 2013) and control devices such as mobile robots (LaFleur et al., 2013) or aircraft (Kryger et al., 2017). Alternatively, passive BCIs, which monitor human cognitive and affective states, can be used to detect drowsiness in car drivers (Picot et al., 2012) or high workload in pilots (Berka et al., 2007) and air traffic controllers (Aricò et al., 2016).

While the first BCIs were inaccurate, cumbersome and time-consuming to apply, advances such as dry (Chi et al., 2010) or water-based (Volosyak et al., 2010) electrodes and new sensor fusion methods (Müller-Putz et al., 2015; Novak and Riener, 2015) have significantly increased both their performance and user-friendliness. Nonetheless, the question remains: just how accurate and reliable are BCIs?

### Previous brain-computer interface competitions

Benchmarking the performance of BCI technology is critical, as it allows researchers to evaluate how different approaches compare to each other (Zander et al., 2011). For instance, benchmarking allows us to determine whether a certain classifier allows intended commands to be more accurately identified from electroencephalographic (EEG) data (Zander et al., 2011). In the past, BCI benchmarking mainly focused on comparing different signal processing and classification methods on the same set of previously recorded EEG data. This was done as part of BCI competitions involving many different researchers. The first such competition was announced in 2001 to a smaller BCI community (Sajda et al., 2003), while follow-up competitions focused on topical challenges—from single-trial EEG classification (Blankertz et al., 2004) to classification of continuous EEG data without trial structures and classification of signals affected by artifacts (Tangermann et al., 2012).

The common feature of all previous BCI competitions was that they performed offline analysis of a previously recorded dataset, allowing different algorithms to be compared using exactly the same raw data. However, when different algorithms are compared offline, they can only perform signal analysis; they cannot perform actions in response to the information extracted from the dataset. For example, if the algorithm determines that the data was recorded when the user wanted to perform a certain action, it cannot assist with this action. The next step is thus to benchmark BCIs online: in a situation where the user sends commands and controls devices using the BCI. This is more challenging than offline benchmarking, as multiple BCI approaches cannot be used online with the same user and same device simultaneously. However, it is critical, as online performance cannot necessarily be predicted from offline performance: as users use a BCI for online control, they learn to compensate for systematic errors and better use the device (Cunningham et al., 2011). Similar issues have been noted in other electrophysiological devices such as myoelectric prostheses (Jiang et al., 2014) and emphasize the need for online benchmarking.

### The goals of the Cybathlon 2016

To fulfill the need for online BCI benchmarking outside the laboratory, we established a BCI competition as part of the Cybathlon—a larger event that aims to both evaluate different assistive technologies as well as showcase them to the general public (Riener, 2016). The first Cybathlon was held in October 2016 in Zurich, Switzerland, and was preceded by a rehearsal in 2015. It consisted of six disciplines: powered arm prostheses, powered leg prostheses, powered exoskeletons, powered wheelchairs, functional electrical stimulation, and, finally BCIs. The overarching aim of the BCI competition was to benchmark different systems in realistic conditions outside the lab using pilots with severe physical impairments. Specific goals were:

- Goal 1: Develop a “benchmark” task that is inherently safe but nonetheless provides a reasonable estimate of how well a given BCI system would perform in a real-world assistive application outside the laboratory.
- Goal 2: Establish a set of benchmarking rules that allow different BCIs to be compared to each other in a fair and safe way, using pilots with disabilities who would be most likely to use such BCIs in everyday life.
- Goal 3: Using results of the 2016 BCI competition, compare different BCI approaches with regard to task performance in order to identify more or less effective approaches.
- Goal 4: Act as an outreach event that increases the general public's interest in BCIs and other assistive technologies.

While the first three goals were purely scientific, the Cybathlon is also a “popular science” event that should be accessible to a broader public. Therefore, the task being performed should be easily understood by an audience of laypersons. For example, commands sent by the BCI should have clearly identifiable consequences in the task. This represents a new type of BCI competition that is less structured and may require different signal processing approaches, but its results would also be more directly applicable to future real-world applications of BCI.

This paper is structured as follows. The “Methods for BCI Benchmarking at the Cybathlon” section presents the Benchmarking methods used in the Cybathlon BCI competition: the task to be performed (Goal 1) and the rules (Goal 2). The “Results of the Cybathlon BCI Competition” section presents the results of the Cybathlon BCI competition as well as how they relate to Goals 3 and 4. The Discussion section then discusses our progress toward all four goals as well as how the rules of the Cybathlon could be modified for future BCI benchmarking either at the Cybathlon or at other events.

## Methods for BCI benchmarking at the Cybathlon

In offline analysis, BCI performance is generally quantified using its classification accuracy (how often the correct desired command is identified from brain activity). However, while classification accuracy is generally correlated with BCI controllability and overall user satisfaction (van de Laar et al., 2013), there is no guarantee that better offline classification accuracy will translate to better online performance (Cunningham et al., 2011). An alternative performance metric is the information transfer rate (how many commands can be sent to a controlled device per minute) (Nicolas-Alonso and Gomez-Gil, 2012), but this metric depends heavily on the context. Instead, we chose to benchmark BCIs at the Cybathlon according to a different metric: the time it takes a user to successfully complete a real-world task using the BCI. The development of an appropriate benchmarking task was Goal 1 of the Cybathlon, and this task is described in the “Benchmark Game” section.

Improvements in BCI performance can be facilitated by improvements at any level of BCI, from hardware improvement to better noise removal and more accurate classification. While we cannot analyze the individual contribution of each BCI component in an online application, it is still important to set rules for each level of BCI (including the human user) to ensure fair comparison of the results between participating teams. Setting these rules was Goal 2 of the Cybathlon; they were first developed prior to the 2015 rehearsal, then modified after the rehearsal following feedback from participating teams. The rules and the justification for them are described in the “Benchmarking Rules” section.

### Benchmark game

#### Concept

As stated in Goal 1, a desirable BCI benchmarking task should represent a realistic challenge for BCI (i.e. similar to an actual assistive application), can be understood by the general public, and is inherently safe. We first considered using a BCI to control a wheelchair, as this is a common application (Carlson and Millán, 2013) and since wheelchairs are already present at the Cybathlon. However, the idea was eventually discarded for two reasons. First, there was a risk of an inaccurate BCI causing unsafe behavior of the wheelchair and injuring the pilot. Second, the additional hardware would increase the uncertainty of the benchmarking process, as it would introduce many variables (e.g., the construction of the wheelchair, the control algorithms) that differ from device to device and are unrelated to the BCI itself. Controlling a mobile robot (LaFleur et al., 2013) or any other remote-controlled device was discarded for similar reasons. To maximize safety and minimize dependence on hardware unrelated to BCIs, we instead chose to use a computer game. Computer games are often used as a demonstration of BCIs, are inherently safe, and would look attractive to a layperson, so their use in the Cybathlon was considered appropriate.

The most intuitive competitive multiplayer game is a racing game, and most other Cybathlon disciplines involve racing over an obstacle course. Therefore, we decided to use a racing game where up to four pilots' in-game avatars can compete either on the same track (e.g., car racing) or on parallel tracks (e.g., horse race, sprint). To save development time and effort, commercially available racing games were first considered for use with BCIs. However, while commercial games are visually attractive and would undoubtedly be well-received by the audience, they are not meant for BCI control, as they require many commands to be sent to the game with split-second precision. By contrast, many state-of-the-art BCIs achieve information transfer rates of only approximately 20 bits/min (Nicolas-Alonso and Gomez-Gil, 2012). A racing game that is slow enough to be controllable with BCIs was thus developed in cooperation between ETH Zurich and the Zurich University of the Arts (ZHdK), Switzerland.

A major concern in the design of the BCI-controlled racing game was that some BCIs at the competition could have a very low accuracy and might be unable to effectively control the game. Therefore, one game design rule was that a racer should eventually reach the finish line even if he/she sends no correct commands—correct commands should speed racers up while incorrect commands should slow them down but not stop them completely. Thus, an “obstacle course” game was created where up to four pilots' virtual avatars run along a track with different types of obstacles. Each obstacle appears at the same spot on the track for all competitors. Sending the correct command through the BCI at the correct time, for example, may make the avatar jump over an obstacle while sending no command or sending the wrong command makes the avatar hit the obstacle and temporarily slow down.

**Figure 1:**
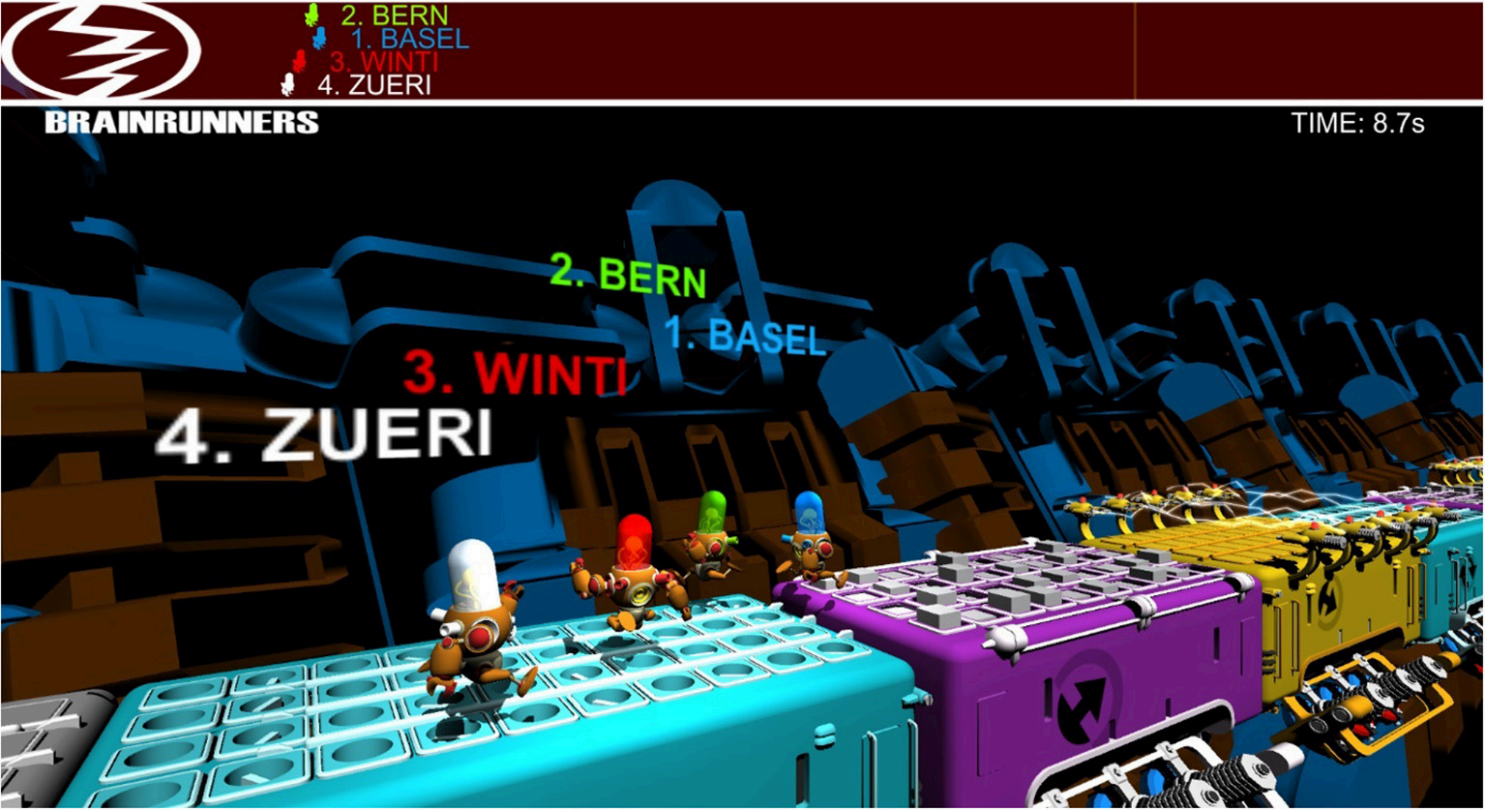
A screenshot of the audience view of the BrainRunners game, which allows up to four avatars to compete on parallel tracks. Each track consists of multiple instances of three different “action” fields colored purple, cyan and yellow (on which the pilot must send the correct command via the brain-computer interface in order to speed up their avatar) as well as gray “no-input” fields (on which the pilot should not send any commands).

To ensure that pilots are not distracted by too many visual features, another design decision was made: different visual displays of the game were provided to pilots and to the audience. Pilots were shown a simplified display that is focused on their own avatar and has no distracting elements (for example, background is removed and textures are simplified). The audience, on the other hand, was shown a more visually rich version of the game. A screenshot of the audience view of the BCI game, which is titled BrainRunners, is shown in Figure 1.

#### Number and type of commands

Assuming different types of obstacles, each pilot's avatar should accept different commands. For example, one command would make the avatar jump while another would make the avatar slide. Each would only be effective for a specific type of obstacle: for example, an obstacle at low height would require the avatar to jump while a high obstacle would require the avatar to slide underneath it. This then raises the question of how many commands can realistically be sent by a BCI. The simplest BCIs only have two output states: “command” or “no command”, which are sent depending on the level of EEG activity (low or high). More complex devices can produce several different commands as well as a “no command” state depending on, e.g., which regions of the brain are active. How can we enable simpler BCIs to participate in the competition while still allowing more complex devices to have an advantage?

The first decision was that the game should operate in asynchronous mode (Mason and Birch, 2000): there should be times in the game when no command should be sent to it, and sending any command should be penalized at those times. Such asynchronous control was a major component of previous BCI competitions (Tangermann et al., 2012), and is commonly used in assistive devices—the user may require assistance at any moment, but there are also times when no assistance is needed (Pfurtscheller et al., 2005; Ortner et al., 2011; Sakurada et al., 2013). For example, a BCI-controlled wheelchair should remain stationary while the user focuses on activities such as eating or working, but should also be ready to receive movement commands at any time. The alternative to such asynchronous control would have been to have the game ignore BCI inputs until the avatar reaches an obstacle, then check if the user has sent the correct command. However, given that assistive devices commonly operate in asynchronous mode, this alternative was unrealistic for online benchmarking.

After settling on asynchronous operation with a “no command” state, we implemented three different commands (“rotate,” “jump,” and “slide”) that the pilot can send via the BCI. Each command has a specific time when it should be used; using it at the correct time gives the pilot a bonus while using it at an inappropriate time penalizes the pilot. However, even if the pilot never sends any command, their avatar will eventually reach the finish line. This allows BCI devices based on 2-class classifiers to compete by only using one command (e.g., “jump”), while developers of more complex BCIs have to consider whether the potential benefits of the other two commands outweigh the potential penalties of using them incorrectly.

As seen in Figure 1, the BrainRunners game allows up to four pilots to compete simultaneously, with their avatars running in parallel. There are four types of fields on the track:

- No-input field (where no command should be sent)—gray in Figure 1,
- spinning winds (where the pilot can send the “rotate” command to speed up and is otherwise slowed down)—cyan in Figure 1,
- stumbling blocks (where the pilot can send the “jump” command to quickly hover over the stumbling blocks and is otherwise slowed down)—purple in Figure 1, and
- sticky lasers (where the pilot can send the “slide” command to quickly slide under the lasers and is otherwise slowed down)—yellow in Figure 1.

These fields can be seen by pilots at least 10 s before their avatar reaches them, giving pilots time to react to upcoming fields and accounting for potentially slow BCI paradigms.

Sending the correct BCI command on its corresponding field causes the pilot's avatar to speed up and run at a higher speed until the end of the field or until another command is sent. This can be done whenever the avatar is on the field; doing it as soon as the avatar reaches the field results in the greatest benefit, but doing it later is still beneficial (as it makes the avatar cross the remaining part of the field faster). On the other hand, sending the incorrect command (or any command on the no-input field) penalizes the pilot by making their avatar slow down. This penalty slowdown can be overridden on an action field by sending the correct command, which will speed the avatar up again. Similarly, if the player first sends a correct command, then an incorrect one, the speed-up will be overridden by the incorrect command. On an action field, any speed-up or slowdown lasts until the end of the field, after which the pilot's avatar returns to the default speed. On the no-input field, the penalty slowdown lasts for a predetermined amount of time, and the avatar can thus return to the default speed before the end of the field. This is because there is no way to correct an incorrectly sent command on a no-input field.

The penalty and override structure ensures that randomly sending all possible commands one after another is not an optimal strategy and is (depending on the weighting of reward and penalty) possibly worse than sending no command at all. This structure mimics a real-life application of controlling an assistive device where a command being executed can be overridden with another command.

Six different parameters are used to balance the game mechanics:

1. Default speed on no-input field if pilot has (correctly) not sent a command: *s*_*default,NoInput*_.
2. Reduced (penalty) speed on no-input field if pilot has sent any command: *s*_*punish,NoInput*_.
3. Maximum penalty (reduced speed) time on the no-input field: *t*_*maxPunish,NoInput*_.
4. Default speed on an action field (rotate/jump/slide) if pilot has (incorrectly) not sent a command: *s*_*default,Action*_.
5. Increased (reward) speed on action field if the correct command has been sent: *s*_*reward,Action*_.
6. Reduced (penalty) speed on action field if a wrong command has been sent: *s*_*punish,Action*_.

All versions of BrainRunners obey the inequality: *s*_*reward,Action*_ > *s*_*default,NoInput*_ > *s*_*default,Action*_ > *s*_*punish,NoInput*_ = *s*_*punish,Action*_. This means that the pilot's avatar has four possible speeds:

- The default medium speed is given on the no-input field if no command is sent,
- the high speed is only given if the correct command is sent on an action field,
- sending no command on an action field results in the low speed,
- sending the wrong command on an action field or any command on the no-input field results in the very low speed. This is done because sending no BCI command should still be a better option than sending the wrong command.

**Figure 2:**
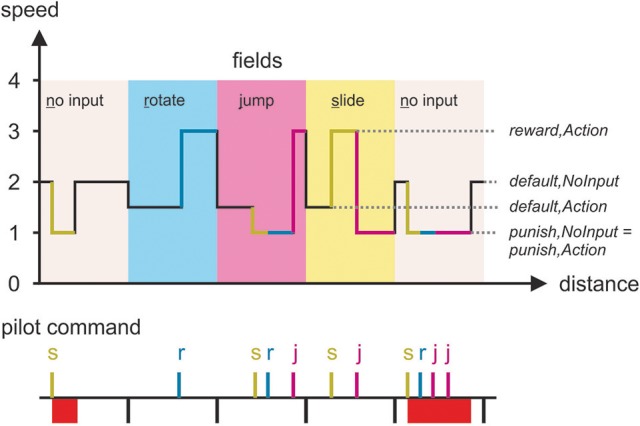
Changes in the speed of the pilot's avatar **(Top)** as the pilot sends different commands (**Bottom**: s, slide; r, rotate; j, jump) to the game on different fields. The 1–4 values are purely symbolic, and exact speeds differ between versions of the game; see main text for values from the 2016 competition.

An example of game speed on different fields in response to pilot commands is shown in Figure 2. In the game version used at the 2016 Cybathlon, the values are as follows: *s*_*reward,Action*_ = 3, *s*_*default,NoInput*_ = 1, *s*_*default,Action*_ = 0.5, *s*_*punish,NoInput*_ = 0.3, *t*_*maxPunish,NoInput*_ = 4. This means, for example, that if the pilot sends any command on a no-input field, the avatar's speed decreases from the default value to 30% of the default value for a punishment period of 4 s. Conversely, if the pilot sends the correct command on an action field, the avatar's speed increases from 50 to 300% of the default value until the avatar crosses the field or a different command is sent. If the pilot sends no command, their avatar needs about 6 s to cross a no-input field and about 11 s to cross an action field. The exact values of the different speeds and the maximum penalty time were not made public prior to the 2016 Cybathlon and could only be obtained indirectly by practicing with the training version of the game. This was done since a real-world application would not have a perfectly definable cost/reward structure for BCI control. The values were balanced so that a realistic BCI accuracy achieves a better result than sending no command at all, which in turn achieves a better result than randomly sending commands. For future competitions, we intend to change the different values in order to provide participating teams with a new challenge.

A last balancing factor is the prevalence of the different fields. Each action field (rotate/jump/slide) must have the same number of appearances in the race. This ensures that teams that use fewer than three commands are indifferent as to which one they omit. Furthermore, the ratio of no-input vs. action fields has a direct influence on how important it is to avoid false positives or false negatives. In the final version of the game, there are four instances of each action field as well as four no-input fields, for a total of 16 fields that can appear in different orders. We acknowledge that such a short race is vulnerable to random effects and thus does not necessarily produce unbiased BCI results; however, a short duration was necessary since the Cybathlon BCI race was watched by a live audience of laypersons.

### Benchmarking rules

Goal 2 of the Cybathlon BCI competition was to establish a set of benchmarking rules that allow different BCIs to be compared to each other in a fair and safe way. These rules are described in this section, and include acceptable BCI paradigms, acceptable hardware and software, and inclusion/exclusion criteria for human pilots.

#### Brain-computer interface paradigms

The first, fundamental decision regarding acceptable BCI technology was whether to limit the competition to EEG-based devices. EEG is the most popular BCI modality, and was the focus of previous BCI competitions (Sajda et al., 2003; Blankertz et al., 2004; Tangermann et al., 2012). Other technologies, such as magnetoencephalography and functional magnetic resonance imaging, were excluded simply because they are not portable. Furthermore, invasive (e.g., implanted) BCI technologies were excluded since it would be difficult to find sufficient human pilots with implanted BCI devices and since pilots with noninvasive devices may potentially be at a disadvantage compared to pilots with implanted devices. In the end, we allowed BCIs based on EEG as well as those based on functional near-infrared spectroscopy. Functional near-infrared spectroscopy has been previously used in BCIs (Sitaram et al., 2007; Zimmermann et al., 2013) and can even be combined with EEG to improve performance (Fazli et al., 2012). However, all teams used only EEG-based BCIs.

Within EEG-based BCIs, there are several paradigms for how the user can use their brain activity to control a device. To decide which paradigms to permit, our guideline was that the BCIs should do what the general public believes they do: read internal thought processes. In what proved to be a somewhat controversial decision, we thus forbade BCIs that require additional external stimuli (e.g., a second screen). Principally, this excluded BCIs based on SSVEPs and visually evoked P300 waves. For example, SSVEP-based BCIs provide the user with a dedicated screen, and different parts of the screen flash at different frequencies. The user selects a desired BCI command by looking at its corresponding part of the screen, and the BCI recognizes the command by measuring the dominant frequency of the visual cortex's EEG activity (Middendorf et al., 2000; Gao et al., 2003; Pfurtscheller et al., 2010; Ortner et al., 2011; Sakurada et al., 2013). Similarly, P300-based BCIs commonly evoke P300 waves via the oddball paradigm: different stimuli (most commonly rows and columns of letters) are successively highlighted on a dedicated screen, and the user exhibits a P300 wave in response to the stimulus of interest (e.g., the letter they wish to write) (Fazel-Rezai et al., 2012). Though SSVEP- and P300-based BCIs are relatively fast and accurate (Nicolas-Alonso and Gomez-Gil, 2012), we feel that the required additional screen is inappropriate for many applications and that, in the case of SSVEPs, the same result (gaze-based selection) can be achieved with a camera-based eye tracker.

Due to the exclusion of paradigms that rely on external stimuli, we expected that the most commonly used paradigms would involve motor imagery, where the pilot generates EEG by imagining limb motions, and/or mental imagery, where the pilot generates EEG by carrying out specific mental tasks such as arithmetic (Obermaier et al., 2001; Friedrich et al., 2012). This was indeed the case at the Cybathlon 2016, as described in the “Results of the Cybathlon BCI Competition” section; however, other potentially valid approaches have been proposed. For example, an anonymous reviewer of this paper proposed using error-related potentials (Chavarriaga et al., 2014), which are evoked by external stimuli provided by the game itself (e.g., seeing the pilot's avatar slow-down in response to an incorrectly sent command). In future BCI races, we would consider the use of error-related potentials (and other brain activity evoked by the game itself) to be acceptable since it does not require any stimuli that are not already present in the application.

#### Hardware, skin preparation, and signal sites

To capture low-amplitude EEG signals, it is critical to use multiple electrodes (ranging from 4 to 64 in state-of-the-art systems Nicolas-Alonso and Gomez-Gil, 2012) with a high signal-to-noise ratio. For the Cybathlon, the primary rules regarding electrodes were that they should be safe for human use and should not penetrate the skin, though hair removal and light skin abrasion at the electrode sites were permitted. As long as this rule was met, we permitted both wired and wireless electrodes, and placed no restrictions on the use of gel—gel-based, water-based (Volosyak et al., 2010) and dry electrodes (Guger et al., 2012) were all acceptable.

The electrodes are connected to signal amplifiers that generally compromise between bulkiness and signal quality. The main Cybathlon rule for amplifiers, again, was that they should be safe for human use; no restrictions were placed on, for example, the use of wireless amplifiers. Teams using commercial devices were asked to provide the manufacturer's statements of conformity while teams that wanted to use their own (built in-house) devices were required to conduct a risk analysis and provide a full report. All documentation was reviewed by two independent BCI experts, who were provided with a checklist of safety items (e.g., surge protection) and could note outstanding issues to be checked in person prior to the competition. This checklist is provided in Appendix I (Supplementary Materials). However, these safety issues were minimal, as all teams used commercially available devices rather than in-house prototypes.

Finally, all EEG electrode sites (frontal, central, parietal etc.) were permitted. While some areas are known to be more closely related to, e.g., motor or mental imagery, we saw no need to limit teams to those areas. Indeed, most teams chose to use a large number of evenly spread electrodes so that they could perform spatial filtering on the data.

#### Artifact removal and classification

EEG signals have amplitudes in the microvolt range and are vulnerable to different artifacts. For instance, signals from frontal areas could be contaminated by electrooculographic (EOG) artifacts while signals from parietal areas could be contaminated by neck muscle activity (Delorme et al., 2007). Thus, EEG signals are almost always filtered to remove noise and artifacts prior to further analysis (Ramoser et al., 2000; Blankertz et al., 2008). However, unscrupulous participants could also make use of artifacts to unfairly boost the performance of their BCI system: for instance, since blink artifacts have a high amplitude compared to actual EEG, a team could potentially train their pilot to blink in order to send a command.

While we did not expect intentional cheating, we did request that teams provide us with a description of their artifact removal procedure. As EOG was considered to be the most problematic potential source of artifacts, we also requested that teams include EOG recordings in their setup. This way, judges could check whether eye artifacts have been adequately removed from EEG. As an alternative to a description of each team's artifact removal procedure, we briefly considered simply implementing standard artifact removal software and requiring each team to use the same software. However, while this would make it much easier to monitor the teams, it would also require extensive testing to ensure that the artifact removal software is compatible with all teams' systems. Furthermore, as artifact removal is an important part of ensuring real-world BCI performance (Fatourechi et al., 2007), we wanted to encourage teams to develop novel artifact removal methods.

Once EEG signals have been filtered to remove noise and artifacts, useful features need to be extracted from the signals, and classification algorithms need to be applied in order to turn the extracted EEG features into discrete commands that are sent to the controlled device (Bashashati et al., 2007; Brunner et al., 2011; Nicolas-Alonso and Gomez-Gil, 2012; Müller-Putz et al., 2015; Novak and Riener, 2015). For online performance, asynchronous operations represent a particular challenge: the BCI should allow the user to perform a command at any time, but should also remain idle most of the time when the user does not desire to use the BCI (Mason and Birch, 2000; Pan et al., 2013; Williams et al., 2013). No restrictions were placed on feature extraction and classification, as we again wanted to encourage teams to develop novel classification methods.

#### Inclusion and exclusion criteria for human pilots

The final part of planning the Cybathlon's BCI benchmarking process was to define the type of people that will operate the BCI. A few generic requirements were set: pilots should be at least 18 years old, should understand the competition, and should not suffer from epilepsy or cybersickness. This would be sufficient for a general benchmarking competition; however, the Cybathlon focuses on assistive technologies, so the pilots should be drawn from the population that would use such technology.

The main target population for assistive BCIs are severely paralyzed people who cannot use their limbs to operate technology. For example, they could be used by tetraplegics to control wheelchairs (Carlson and Millán, 2013). However, it was difficult to define the minimal acceptable level of disability. Should participation require tetraplegia or only paraplegia? And should the disability be due to a specific cause (e.g., spinal cord injury)? This was primarily a question of fairness, as there is evidence that neural representation of different thoughts differs between spinal cord injury and brain injury survivors (Murphy and Corbett, 2009), that BCI classification accuracies differ between tetraplegics and paraplegics (Müller-Putz et al., 2014), and that brain activation after injury is affected by the degree of clinical recovery (Kokotilo et al., 2009).

Several solutions were considered: allowing only tetraplegia, allowing both tetraplegia and paraplegia, and having separate BCI subdisciplines for severe and lighter impairment. At the 2015 rehearsal, we permitted teams to use unimpaired BCI pilots if they were unable to field a pilot with motor impairment. After the rehearsal, the inclusion and exclusion criteria were finalized: pilots should be tetraplegic or tetraparetic (American Spinal Cord Injury classification levels A to C) due to any injury, with each pilot's eligibility determined individually. This requirement was chosen since all teams at the rehearsal agreed that tetraplegic pilots represent the most promising target population for assistive BCIs. No restrictions were implemented with regard to available sensory channels (e.g., damaged sensory nerves).

To ensure ethical integrity with regard to involvement of human pilots, the Zurich Cantonal Ethics Commission was consulted about the Cybathlon (request no. 2016-00161). The Commission ruled that, as an event, the Cybathlon was exempt from ethics approval since it was considered to be primarily an exhibition and outreach event rather than a fundamental scientific study. Nonetheless, informed consent was obtained from all participating pilots, and participating teams were advised to obtain ethics approval from their own institutions if required for BCI training.

## Results of the Cybathlon BCI competition

### Event schedule

Both the 2015 rehearsal and 2016 Cybathlon began with two inspections: a technical check of the hardware and software by independent BCI experts (to ensure safety and general adherence to the rules) and a medical check of each pilot's health status by a team of physicians. After that, qualification races were performed, followed by two final races (A-final and B-final).

There were three qualification races, with four teams participating in each race. As previously described, each race contains four instances of each action field as well as four no-input fields, for a total of 16 fields. All three qualification races had these 16 fields appear in different orders that were not known to participants in advance. In each race, the time needed for each team to reach the finish line was measured. The four fastest teams from all three races advanced to the A-final while the next four teams (ranked 5–8) advanced to the B-final. Each race lasted approximately 3 min, though moving the teams to and from the competition stage took approximately 15 min per race. Again, we acknowledge that such short races were likely influenced by random factors and may not have captured the best performance of each BCI technology; however, the need for short and attractive races was dictated by the live audience of laypersons.

### Team approaches and results

Ten teams participated in the 2015 rehearsal. They were affiliated with the Technical University of Graz (Austria), INSEP Paris (France), Technical University of Darmstadt (Germany), University of Padova (Italy), Ecole Polytechnique Federale de Lausanne (Switzerland), Mahidol University (Thailand), University of Essex (United Kingdom), Gray Matter (United Kingdom), University of Ulster (United Kingdom), and University of Houston (USA).

Eleven teams then participated in the 2016 competition, though there were significant changes from the 2015 lineup: some teams dropped out after the rehearsal because they could not find a tetraplegic pilot (required in 2016 but not 2015) while other teams either signed up after the rehearsal or had chosen not to participate in the rehearsal. Tables 1–**3** present a list of the participating teams at the 2016 competition, as well as a brief overview of the teams' hardware (Table 1), software (Table 2) and pilots (Table 3). While information about all teams' approaches was collected by the technical experts of the Cybathlon, it was originally kept confidential. After the competition, we asked teams if they would provide us with a brief publishable summary of their approach; Tables 1–3 contain information for those teams that provided a summary.

**Table 1:** The teams of the 2016 Cybathlon Competition and the hardware they used.

**Team name/affiliation**	**Race time (s) qualifier**	**Race time (s) finale**	**Signal amplifier**	**Electrode number, locations**	**Electrode type**	**Prep time**
Brain Tweakers/CNBI EPFL^*^	90	190	g.tec g.USBamp	16, mainly frontal and central	Gelled, active	5–10 min
Brain Tweakers/CNBI EPFL^*^	123	125	g.tec g.USBamp	16, mainly frontal and central	Gelled, active	5–10 min
BrainGain/Radboud University	135	156	TMSi Mobi	24, evenly spread	Gelled, passive	20 min
BrainStormers/University of Essex	146	161	BioSemi ActiveTwo	64, evenly spread	Gelled, active	15 min
Athena-Minerva/TU Darmstadt	148	146	BrainVision actiCHamp	128, evenly spread	Gelled, active	30–60 min
OpenBMI/Korea University	149	149				
Neurobotics, Russia	161	132	Neurobotics			
NeuroCONCISE/University of Ulster	165	136	g.tec g.Nautilus	16, evenly spread	Gelled, active	15 min
Mahidol University	167	not in final	g.tec g.USBamp			
Ebrainers/Pazmany Peter Catholic University	186	not in final	BrainVision actiCHamp	32, evenly spread	Gelled, active	15 min
Mirage91/TU Graz	196	not in final	g.tec g.USBamp	32, mainly sensorimotor areas	Gelled, active	20 min
Ecole Supérieure de Lyon	none^**^	not in final	g.tec g.USBamp	16, evenly spread	Gelled, active	5–10 min

*Teams are ranked according to the time they needed to complete the qualifier race. The top four teams from the qualifier participated in the A-final while the next four participated in the B-final. Blank spaces in equipment columns indicate the team did not provide this data*.

* *The Brain Tweakers team participated with two pilots, both of which used the same hardware and software*.

** *The pilot of the Lyon team did not pass the medical check and thus did not participate in the qualifier race*.

**Table 2:** The teams of the 2016 Cybathlon Competition and the software they used.

**Team name**	**Filtering**	**Imagery used for commands**	**Feature extraction and classification**
Brain Tweakers	Bandpass, notch, spatial	Hands, feet, hands+feet	Power spectral density, feature selection with canonical variate analysis, classification using bayesian maximum a posteriori probability equivalent to qda
BrainGain	Detrend, common average reference, spatial whitening, bandpass	Left hand, foot, tongue	Power spectral density, regularized logistic regression
BrainStormers	Bandpass, spatial	Left hand, foot, word concatenation, auditory	Common spatial patterns, power spectral density, classifier ensemble
Athena-Minerva	Bandpass, spatial	Hand, word association, mental arithmetic	Power spectral density, multiple binary classifiers
NeuroCONCISE	Neural-time-series-prediction-preprocessing, subject-specific bandpass, common spatial patterns	Left hand, right hand, feet	Log variance of filtered signals, linear discriminant analysis and multiple binary classifiers, postprocessing of classifier output
Ebrainers	Bandpass, spatial and temporal filtering	Hand, feet, mental arithmetic	Power spectral density, phase locking index, complex covariance patterns
Mirage91	Bandpass	Hand, feet, mental arithmetic, auditory	Common spatial patterns + slda, adaptive classifier
Ecole Supérieure de Lyon	Spatial, independent component analysis	Left hand, right hand, tongue	Power spectral density, common spatial patterns, support vector machine

*Reported only for teams that agreed to make this information public. All teams used imagery (motor or otherwise) to generate BCI commands*.

**Table 3:** Characteristics of the pilots at the 2016 Cybathlon Competition.

**Team name**	**Pilot year of birth**	**Type of injury**	**Year and cause of injury**
Brain Tweakers (winner, qualifier)	1968	SCI at C5/C6 with ASIA A	1989, traffic accident
Brain Tweakers (winner, finale)	1986	SCI at C5/C6 with ASIA A	2003, bike jumping accident
BrainGain	1960	SCI at C5-7 with ASIA A	1982, bleeding in blood vessel
BrainStormers	1963	SCI at C5/C6 with ASIA B	1987, diving accident
Athena-Minerva	1989	SCI at C5/C6 with ASIA B	2008, traffic accident
OpenBMI	1968	SCI at C4/C5 with ASIA C	1995, traffic accident
Neurobotics	1982	SCI, details not publicly disclosed	2002, diving accident
NeuroCONCISE	1971	SCI at C5 with ASIA A	1993, traffic accident
Mahidol University	1989	SCI at C4 with ASIA A	2006, gunshot
Ebrainers	1989	SCI at C6 with ASIA B	2006, sports accident
Mirage91	1979	incomplete locked-in syndrome	2014, brain stem infarction and cerebellar infarction
Ecole Supérieure de Lyon	1980	SCI at C5 with ASIA B	2012, traffic accident

*SCI, Spinal cord injury; ASIA, American Spinal Injury Association impairment scale*.

As can be seen from Tables 1, 2, the best approach is difficult to identify. There was no clear advantage to using a particular amplifier, a particular number of electrodes, or particular electrode locations. Most teams used the g.USBamp (g.tec Medical Technologies GmbH, Austria) or BrainVision actiCHamp (Brain Products GmbH, Germany) amplifiers and their associated electrodes, but this was partially for nonscientific reasons: g.tec and Brain Products were sponsors of the Cybathlon, and agreed to provide free BCI hardware to several participating teams. Still, it is interesting that all teams used gelled electrodes, and may suggest that dry electrodes are not yet suitable for use outside the lab; however, due to lack of data, this is only speculation. Furthermore, while some teams originally expressed an interest in consumer-grade EEG devices from Neurosky (USA) and Emotiv Systems (Australia), none participated with such devices.

Similarly, there is no clear advantage to any software approach, and many teams had similar approaches (e.g., use of spatial filtering and power spectral density based features). This use of similar approaches was likely because we forbade the use of additional external stimuli, which prevented teams from using other approaches such as SSVEPs or visually evoked P300 waves. There is also no clear advantage due to pilot age or injury severity (Table 3), and we believe that Goal 3 was not successfully fulfilled—we were unable to identify any factors that clearly improve BCI performance. We believe that future competitions should examine the effect of other human factors on performance, as explored further in the Discussion section.

### Spectator feedback and scope of outreach

The Cybathlon was highly effective from an outreach perspective. In addition to coverage from Swiss news agencies, 140 media representatives from 15 countries registered to cover the event. They included representatives from, e.g., Al Jazeera English, BBC, Bloomberg News, CNN, Die Zeit, ORF, Reuters, Wired Japan, and many more. Over 300 articles were published about the Cybathlon in 2016. Furthermore, the Cybathlon website (www.cybathlon.ethz.ch) was visited by approximately 47,000 different people in October 2016 alone, and approximately 4,800 people from all over the world watched the Cybathlon live via the stream on the Cybathlon website. Finally, the Cybathlon trailer has been viewed over 152,000 times on Youtube (as of November 2017).

The Cybathlon was attended in person by more than 4,600 spectators; of those, 283 participated in a brief survey about their experience. The majority (54%) were between 19 and 30 years old, and an additional 38% were between 31 and 60 years old. 70% were from Switzerland, 25% were from other European countries, and 5% were from outside Europe. 51% attended due to an interest in research and technology while 27% attended due to an interest in medical topics. When asked if the Cybathlon fulfilled their expectations, 44% said that their expectations were fully fulfilled and 47% said that the event exceeded their expectations. Thus, we believe that we successfully generated significant public interest, especially among young people with an interest in technology.

The above data was collected for the entire Cybathlon event, regardless of discipline. In addition to that, spectators were also asked what their favorite discipline was. This result was less encouraging: only 9% chose BCIs as their favorite among the six disciplines (as opposed to, e.g., 33% for wheelchairs and 20% for lower limb prostheses). Spectators commented that BCIs were not as attractive as other disciplines because they involved no physical movement—only pilots motionlessly playing the benchmarking game with their mind. Furthermore, some spectators still had difficulty understanding the cause-effect relationship between the BCI and the in-game actions without the help of the announcer, and requested live commentary of the game in multiple languages.

### Feedback from teams and Cybathlon staff

The participating teams and the Cybathlon volunteers also provided feedback about how future BCI competitions could be improved. First, the teams emphasized the need to keep locker rooms close to the stage and carefully temperature-controlled. This was because teams needed to set up the EEG cap in the locker room, then wait for the competition and move the (usually wheelchair-bound) pilot through the building to the stage; reducing the distance between the stage and locker room also reduces the need to readjust the EEG cap on stage.

Second, monitoring the pilots and ensuring fair play proved to be a significant challenge. While we asked the teams to provide access to EEG and EOG recordings for the judges, it was practically impossible to obtain enough expert judges to truly monitor all recordings during the competition. We limited ourselves to watching for voluntary limb movement and excessive eye movement, but this also was not trivial. Impaired pilots could make involuntary motions (e.g., spasms), and it was difficult to draw the line between normal and excessive eye activity. At the rehearsal, one team was warned after the qualification round since their pilot appeared to be blinking very often; however, such assessments are subjective and cheating can be difficult to prove.

Nonetheless, despite challenges in monitoring the pilots, both the 2015 rehearsal and 2016 competition proceeded smoothly. All teams were able to set up successfully, and no BCIs failed to function. Furthermore, the teams agreed that the game was a good testing ground for online BCIs, with three commands and a “no input” state being realistic.

## Discussion

Based on our experiences, we believe that Goals 1, 2 and 4 of the Cybathlon were successfully achieved while Goal 3 was not fully achieved. Sections Goal 1: Benchmarking game, Goal 2: Rules and inclusion criteria, Goal 3: Identifying effective BCI approaches, and Goal 4: Outreach discuss each individual goal in more detail. The discussion then concludes with a few words of advice to teams who may be interested in participating in the next Cybathlon BCI competition (section Advice to future Cybathlon competitors) as well as a few thoughts about the organizational aspects of the Cybathlon BCI competition (section Importance of organizational aspects).

### Goal 1: benchmarking game

We successfully developed a benchmarking game that allowed BCI performance to be measured via task completion time, and the teams agreed that the game was an appropriate stand-in for actual assistive technologies without the danger present in actual assistive devices. Similarly to our game, actual assistive technologies require users to choose among a small number of possible BCI commands, such as left/right for wheelchairs (Carlson and Millán, 2013), walk/idle for robotic gait orthoses (Do et al., 2013), left/right/both/none for BCI-controlled artificial arms (Onose et al., 2012) and so on. As in our game, the command must be sent at an appropriate time in order to avoid undesired consequences such as collisions. Furthermore, an incorrectly sent command can be overridden by a correct one—for example, if a BCI-controlled wheelchair is told to move to the kitchen, this command can be overridden by a command to move to the living room. Finally, the game was played in a less structured setting with many possible distractors and stressors, thus more closely approximating real-world conditions compared to a laboratory.

One change that could easily be made to the game would be to make some fields more common than others—for example, to have 90% of the fields be “no-input” fields. This would likely be a more realistic approximation of an assistive BCI, as BCIs are expected to spend most of their time idle (Mason and Birch, 2000), and not all assistive actions would be required equally often. This would also force teams to decide, for example, which paradigm (e.g., motor or mental imagery) to assign to which command. We originally chose to have all three actions equally probable so that BCIs with only one possible command could still compete; however, given that all teams at the 2016 Cybathlon used a 3-command BCI, we believe that this modification would be very useful in future BCI competitions.

### Goal 2: rules and inclusion criteria

Most of the rules of the Cybathlon BCI race were not controversial, but it is worth briefly discussing acceptable BCI paradigms and inclusion/exclusion criteria. With regard to BCI paradigms, we excluded approaches that require additional external stimuli: SSVEPs, visually evoked P300 waves and others. A few participating teams complained that they felt constrained by the exclusion of these paradigms, and future real-time benchmarking approaches could consider including all BCI types in order to maximize potential impact. This may, however, require a different game design: a SSVEP-based technology would require the pilot to divide their attention between the game and a secondary visual display, but would also support more than 3–4 commands and would allow faster gameplay due to higher information transfer rates (Nicolas-Alonso and Gomez-Gil, 2012).

Furthermore, with regard to pilot inclusion criteria, we focused on tetraplegic pilots since the Cybathlon is meant to be a competition for people with disabilities who use cutting-edge assistive technologies. Future online benchmarking events, however, should choose the pilot inclusion/exclusion criteria based on the characteristics of the target population. A general BCI benchmarking competition could incorporate unimpaired adults with a similar age and level of BCI experience in order to minimize intersubject variability. A competition focused on, for example, P300/SSVEP-based spelling devices, on the other hand, may recruit only tetraplegics and people with locked-in syndrome since these are the ones most likely to use spelling devices (Li et al., 2008; De Vos et al., 2014). Finally, if the goal of a competition is to test generalizability to a wide range of people with disabilities, we could even ask multiple pilots (with different disability levels) to test the same device.

### Goal 3: identifying effective BCI approaches

The greatest weakness of the Cybathlon BCI competition was that we were unable to identify any factors that had a clear effect on BCI performance: there was no clear difference between different hardware and software approaches with regard to the race results. Furthermore, there was no clear effect of the measured pilot characteristics (e.g., pilot age, ASIA A vs. ASIA B injuries). This was partially likely due to the small sample size, as it is difficult to find clear effects based on 11 competing teams. Furthermore, the fact that some newer EEG technologies (e.g., dry electrodes) were not present at the Cybathlon may imply that these technologies are not yet ready for widespread use. Nonetheless, we cannot consider Goal 3 to have been successfully completed. In future competitions, this goal could be achieved more successfully by collecting more data about factors that affect BCI performance and/or using additional BCI performance metrics.

#### Factors that affect BCI performance

For future BCI competitions, we recommend not only increasing the number of pilots, but also obtaining more information about the pilots. For example, BCI performance tends to improve as users train with the system (Neuper and Pfurtscheller, 2010; Lotte et al., 2013), and there was significant variability in the amount of time that Cybathlon pilots spent training with their BCI system, from days to months. Furthermore, different user personalities and cognitive profiles may be more effective at controlling BCIs (Hammer et al., 2012; Jeunet et al., 2016), and pilots with higher motivation may be more effective as well (Sheets et al., 2014). None of these factors were evaluated at the Cybathlon, but future BCI races could capture and analyze them by asking pilots about the time they spent training as well as, for example, using personality and motivation questionnaires. Some data on this topic was reported by the winning team (Brain Tweakers), which fielded two pilots: in the months leading up to the Cybathlon, the pilot who eventually won the qualifier race completed 183 practice races while the pilot who won the final race completed 57 practice races against computer opponents (Perdikis et al., 2017).

In addition to training time and pilot characteristics, another human factor should be investigated in more detail: the strategies that pilots develop to deal with the game. For example, each pilot must decide on their own whether to attempt sending a BCI command on an action field as early as possible (and thus risk sending the command too early, resulting in penalties) or to wait until the avatar is comfortably on the field (thus reducing the potential benefit). Several pilots told us that they had developed their own strategies, and the winning team emphasized that it was critical for the pilot and BCI to adapt to each other (Perdikis et al., 2017). However, information about this factor was not recorded in detail, and we thus do not reliably know how it affected pilots' scores.

Other factors that may have contributed to BCI performance include for example, electromagnetic noise in the environment and sudden distractions in the arena. Such factors could be averaged out by holding multiple races. This would be a suitable solution for in-house evaluations of BCIs by their developers, but was not appropriate for the Cybathlon, which was broadcast on television and over the Internet, necessitating a single exciting race. The winning team compensated for possible distractions in the arena by carrying out mock races in the lab with loud spectators (Perdikis et al., 2017), and we believe that training in uncontrolled environments is essential for optimal BCI performance in any real-world setting.

#### Additional BCI performance metrics

We used only a single BCI performance metric: the time needed to complete the race. However, a more detailed analysis of performance could be achieved using more comprehensive measurements of BCI behavior. For example, future races could measure the total number of incorrectly sent commands, the time needed to successfully send a command on an action field, and the time needed to correct an incorrect command (i.e., the time between an incorrectly sent command and a follow-up correct command on the same action field). By comparing these metrics between teams, such an analysis could determine whether some BCI approaches are, for example, slower or more prone to incorrect commands than others.

### Goal 4: outreach

Based on the feedback obtained from spectators and the high media profile of the Cybathlon, we believe that we were successfully able to showcase BCI technologies to a general public that has, with very few exceptions, only seen it in science fiction movies, books and news articles. Admittedly, not all reactions were positive: as mentioned in section Spectator feedback and scope of outreach many spectators did not find the BCI discipline as attractive as other Cybathlon disciplines (e.g., powered wheelchairs) due to the lack of physical movement and the difficulty relating the BCI to in-game commands. Furthermore, some were surprised by the low accuracy and slow speed of state-of-the-art BCI systems.

While future Cybathlon events will take steps to make the BCI competition even more accessible to laypersons (by, for example, providing more detailed live commentary), we believe that it is very important to provide spectators with a realistic picture of the technology: not only its advantages, but also its disadvantages. This balanced presentation helps the public understand not only the potential of the technology, but also its current state and opportunities for improvement. Furthermore, it encourages developers to take steps to improve BCI performance outside the lab, bringing the technology closer to practical, real-world use.

### Advice to future Cybathlon competitors

As the next Cybathlon is already planned for 2020, we wish to provide a few suggestions to teams interested in participating. Admittedly, these suggestions are purely subjective and based only on our qualitative observations of the 2015 rehearsal and 2016 competition; nonetheless, we believe that they are worth considering.

First, it is critical to find an appropriate pilot early: most of the dropout from the 2015 rehearsal to the 2016 competition was because teams could not find a tetraplegic pilot. Finding an appropriate pilot early also allows training to start early, providing critical BCI experience to the pilot and allowing classification algorithms to be tailored to the pilot. Second, teams should not only aim to improve offline classification accuracy, but should work with the pilot on online testing of the BCI and developing effective strategies for online BCI control (e.g., teaching the pilot how to compensate for possible systematic errors). These points were also emphasized as critical by the winning team (Perdikis et al., 2017). Third, we encourage teams to develop BCI approaches not based only on motor imagery. For example, a combination of motor and mental imagery (used by some of our 2016 teams) could be combined with detection of error-related potentials to provide immediate error correction.

### Importance of organizational aspects

Finally, we wish to briefly comment on a few additional organizational aspects of the BCI competition: the pilot health checks, the technical safety inspections, and judging the competition for fairness. The Cybathlon 2016 organizational structure was such that all six disciplines had roughly the same technical safety inspections, which may have been excessive for BCIs. BCI systems are safer than the technologies used in the other disciplines, which can seriously injure the user (e.g., via the powerful motors of a robotic wheelchair), and all teams used commercially available hardware (unlike, e.g., the wheelchair discipline, where most devices were prototypes). Thus, future BCI competitions may consider a more streamlined safety check—for example, skipping the hardware check for all cases where common commercial amplifiers are used. We do consider pilot health checks, however, to be important and appropriate: though BCI devices are relatively safe, the pilots were tetraplegic and thus potentially at more risk than pilots in other disciplines (who were either paraplegic or amputees), so it was necessary to be aware of any possible health issues.

Judging the competition for fairness was expected in advance to be difficult, as we did not have the resources to truly monitor all pilots and collected signals during the competition, and even checking for possible voluntary/involuntary movement is not trivial (see section Feedback from teams and Cybathlon staff). One way to address this issue would be to require teams to record both EOG and the electromyogram (EMG) of different muscles during the event, then have judges check the recordings after the race and disqualify competitors later if necessary. This option was considered before the Cybathlon, but ultimately abandoned since it would be prohibitively time-consuming for the small organizing team to manually check all EOG/EMG recordings and cross-reference them with EEG, BCI outputs, and in-game events to determine whether cheating took place. As an alternative, future events could use a “peer review” method where each participating team leader would be randomly assigned to monitor another team during the race, ensuring fairness. A member of the Cybathlon organizing committee (who would be independent of the participating teams) would then be responsible for resolving any issues noticed by these peer reviewers. However, given that we do not expect teams to intentionally try to cheat during the competition, it is unclear whether such steps are truly necessary.

## Conclusion

Having successfully concluded the Cybathlon 2016, we are confident that our BCI race represents a valid method of benchmarking BCIs online and is a reasonable approximation of actual assistive BCI applications. Furthermore, it provides an easily quantifiable outcome metric (the time needed to complete the task) that reflects both the technical quality of the system as well as the skill of the human user. Finally, different use scenarios can be simulated by changing the game parameters—for example, by changing how often each command is required, how often the user needs to remain idle, and what the penalty for failure is.

Our procedure could be used for online BCI benchmarking in academic and commercial settings. While our race involved tetraplegics, EEG and motor/mental imagery, it could also be modified for other user groups and BCI paradigms. This would allow the advantages of different hardware and software approaches to BCI to be evaluated for many applications. Furthermore, by collecting data about the human user, future benchmarking procedures could examine the influence of both technological and human factors. In this way, the scientific community will obtain a complete picture about how different factors affect online BCI performance in different populations, paving the way for broader adoption of BCIs in many applications.

Finally, as the Cybathlon was streamed over the Internet and recorded by camera teams from all over the world, it has the potential to showcase this technology to a general public that has only seen it in science fiction movies. The Cybathlon has received extensive support, and will become a recurring event, with a Cybathlon 2020 already in planning stages and smaller spin-off competitions foreseen around the world.

## Author contributions

DN is an executive board member of the Cybathlon, led the development of most BCI discipline rules, served as a judge and technical expert at the 2015 and 2016 Cybathlon BCI race, and led the manuscript writing process. RS and DW are co-directors of the Cybathlon, and supervised all six disciplines. NG was the head organizer of the BCI race at the 2015 rehearsal and 2016 Cybathlon. RB and UG led the development of the BrainRunners game. Finally, RR is the initiator of the Cybathlon, and was responsible for high-level organization of all the disciplines.

### Conflict of interest statement

All authors were part of the organizing team of Cybathlon 2016. Though the Cybathlon is a nonprofit event, the authors should not be considered independent observers of the event. The reviewer LT and handling Editor declared their shared affiliation.

## Abbreviations

BCI: Brain-Computer Interface
EEG: Electroencephalogram
EMG: Electromyogram
EOG: Electrooculogram
SSVEP: Steady-State Visually Evoked Potential.
