# Supplementary material for: Benchmarking Brain-Computer Interfaces Outside the Laboratory: The Cybathlon 2016
Source: Front Neurosci. 2018 Jan 11;11:756. doi: 10.3389/fnins.2017.00756 (PMC5768650; doi:10.3389/fnins.2017.00756)
Supplement: Supplementary file 1 [file DataSheet1.docx]

**Appendix I: The BCI safety checklist**

## Documentation Provided by Team

The head of discipline checks if the team has provided the documentation that is needed to do the examination. The following documentation is required:

***All devices***

- Video of the device showing its functionality
- Photo of the device

***For commercial devices***

- Declaration of Conformity
- Technical documentation (e.g. manual; design, usage of the product)
- Records of how the product conforms to relevant European standards
- European Community type-examination certificates (if applicable)
- Confirmation that the device(s) will be used at the event according to the specified standards (as intended). Otherwise the *Risk Management File* is required.

***For prototypes or modified commercial devices***

- Risk Management File (please sign)
- Information about batteries (usage, charging, temperature, etc.)
- Residual risks relevant to power failure or shutdown
- Maintenance instructions
- Start-up and restarting instructions
- Continuous usage instructions

## Checklist

**Electrostatic discharge (ESD) protection is sufficient?**

*ESD protection is sufficient or personal protective equipment is required by the pilot or any nearby person – The team provides the necessary protective equipment (IEC 61000-4-2 gives additional guidance).*

**No exposure to harmful substances/materials?**

*Contact with harmful substances/ Leakage or fluid emissions from the device (e.g. hydraulic fluid) is avoided; no materials that might cause allergies should be used at a surface which comes into contact with human skin (e.g. nickel, chromium, some types of rubber).*

**No loose wires and cables?**

*No loose wires that could touch a person (open leads) or could be a potential snag hazard for the pilot or any other person in the vicinity of the device. All cables must be routed closely to the device or within the device and must be properly secured (cable ties, clamps, etc.).*

**Maximum voltage does not exceed 50V?**

*According to EN 60601, devices that are operated at voltage levels less than 50V are considered as safe. If a device is operated at higher voltages, it must be ensured that no hazards can be caused by the energy source (e.g. sufficient insulation is existent).*

**Is the pilot able to control the BCI avatar?**

*On the registration day, the connection between the device and the BCI game can be tested.*

**Is the user protected from power surges?**

*There are reliable and well proven mechanisms to protect the pilot from power surges.*
